# Prevalence of human onchocerciasis in Ethiopia: a systematic review and meta-analysis

**DOI:** 10.4314/ahs.v25i2.3

**Published:** 2025-06

**Authors:** Tadesse Hailu, Getaneh Alemu, Megbaru Alemu

**Affiliations:** Department of Medical Laboratory Science, College of Medicine and Health Sciences, Bahir Dar University, Bahir Dar, Ethiopia

**Keywords:** Onchocerciasis, Prevalence, Ethiopia, Onchocerca vulvulus

## Abstract

**Background:**

Onchocerciasis is a vector-borne disease caused by the tissue nematode Onchocerca volvulus. Despite its importance for targeted intervention, the national prevalence is not well addressed in Ethiopia.

**Objective:**

This review aimed to determine the pooled prevalence of human onchocerciasis in Ethiopia.

**Methods:**

All literature published from 1973 to July 2022 were included in the present review A systematic review and meta-analysis was done following PRISMA guideline and checklists. Studies conducted on the prevalence of onchocerciasis in Ethiopia were searched from PubMed, Google Scholar, Scopus, ScienceDirect, and MEDLINE databases. Comprehensive meta-analysis version 2.2 software was used to calculate the pooled prevalence. Heterogeneity between studies was assessed using Cochrane Q test and I2 test statistics based on the random effects model.

**Results:**

Twenty-one studies, which recruited a total of 14,983 participants, were included in the present review. The overall pooled prevalence of Onchocerca volvulus in Ethiopia was 31.8% using the random effect model. The heterogeneity between studies was high and significant (Q = 2881.2, I2 = 99.3%, P-value < 0.001).

**Conclusions:**

The prevalence of onchocerciasis is high in Ethiopia, despite the implementation of prevention and control measures. Therefore, the existing mass drug administration program should be strengthened.

## Introduction

Onchocerciasis, also called river blindness, is a parasitic disease under the neglected tropical diseases caused by Onchocerca volvulus (O. volvulus)^1^. The disease exists in Africa, with some foci in Latin America and Yemen; however, more than 99% of infected people live in 31 African countries. Based on 2017 estimation, there were 20.9 million O. volvulus infections worldwide; 14.6 million of the infected people had skin disease and 1.15 million had sight loss^2^.

In Ethiopia, more than 16.3 million people live in onchocerciasis endemic areas, of which 5 million are infected. The rate of infection in highly endemic areas rose to 80–100% among people with age ≥ 20 years old^3^,^4^. Onchocreciasis is mostly found in South-Western, Western and North-Western parts of Ethiopia and it is endemic in five regions of the country, namely; Oromia, Southern Nations, Nationalists, and Peoples Region (SNNPR), Benishangul-Gumuz, Amhara and Gambella^4^,^5^. The disease prevalence is highest in places that are close to rivers and it drops gradually as one moves further away from the rivers^5^.

Onchocerciasis is transmitted to humans through exposure to repeated bites of infected black flies of the genus Simulium. Signs and symptoms include severe itching, disfiguring skin conditions, and visual impairment, including permanent blindness. The impact of conchocerciasis is high. Reports in 2013 showed that onchocerciasis was responsible for 34,600 disability-adjusted life-years lost^6^. It also causes visual impairment in around 1-2 million people^7^,^8^ and could ecrease life expectancy by 7–12 years of the most socio-economically weakened and debilitated adults^9^. Onchocerciasis is also a common cause of epilepsy. Studies in tropical Africa reported that epilepsy prevalence is increasing at a progressive rate with rising onchocerciasis prevalence^10^.

Community-directed treatment with ivermectin is the core strategy to eliminate onchocerciasis in Africa^2^. In 2012, Ethiopia also launched onchocerciasis elimination program through Mass Drug administration (MDA) with the goal of attaining interruption of onchocerciasis transmission nationwide by 2020 and to be certified by 2025^5^. The treatment coverage for the last five years has been maintained at more than 80%. Despite many years of ivermectin MDA in endemic areas, the transmission of onchocerciasis in many districts remains persistent^4^. Following detection of the first case in Ethiopia in the year 1939^11^, several epidemiological studies have been conducted in the country, and revealed the presence of the disease in different localities with varying levels of endemicity^12^. However, there is a scarcity of comprehensive data showing the national prevalence. Hence, this review tried to address the prevalence of onchocerciasis in the country and across the regions.

## Methods

### Setting

We included studies conducted all over Ethiopia. Ethiopia is located in east Africa at geographical coordinates of 8o N and 38o E^13^. The country is composed of ten regional states and two city administration councils. Altitude of the country ranges from high peaks of 4,620 meters above sea level to a low depression of 148 meters below sea level. More than half of the country lies above 1,500 meters. The western part of the country is known for dense vegetation accompanied by rivers where vector-borne diseases like onchocerciasis are common public health problems^13^,^14^.

### Data sources and search of literature

Articles were searched in PubMed, Google Scholar, Scopus, ScienceDirect and MEDLINE databases using the keywords: “Onchocreciasis” AND “Ethiopia” OR “Onchoceria volvulus” AND “Ethiopia” OR “Onchocerca” AND “Ethiopia”. A manual search was also conducted/span> on all relevant references listed within the articles identified after an initial electronic search. University repositories were assessed online and potential researchers were contacted in order to get access of manuscripts missed in the electronic search. The electronic data search of studies was conducted from December1 to 20, 2022, independently by two reviewers to minimize bias and missing of studies. Studies published from 1973 to 2022 were included in the present review.

### Study selection

**Fig 1:**
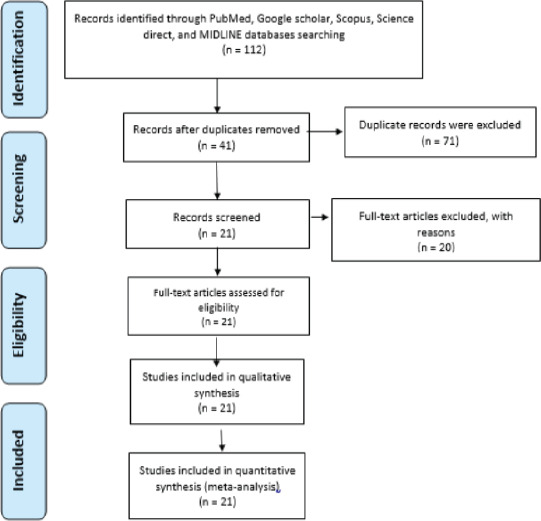
Flow chart showing the selection process of eligible studies

Identification, screening, checking the eligibility, and inclusion of relevant literatures were done following the preferred reporting items for systematic reviews and meta-analyses (PRISMA) guideline and checklist (Figure 1). Both community and facility-based studies published in English language were included in the review. All studies conducted in Ethiopian populations by skin snip examination for O. volvulus infection were included. Peer-reviewed and full text articles were included. All articles dealing with onchocerciasis related knowledge, attitude and practice, clinical signs and symptoms, duplications, review articles and studies including under 5 children were excluded (Figure 1).

### Data extraction

Data were extracted from each study independently by two authors using data collection form constructed in Excel. Information was collected about the year of publication, area (region) of data collection, total number of people participated in the study and infected cases.

### Statistical analysis

The pooled prevalence of onchocerciasis all over Ethiopia and subgroup prevalence across regions, among male and female participants as well as before and after the start of the national control program was computed using comprehensive meta-analysis version 2.2 software (Biostat Inc., Englewood, NJ, USA). Pooled prevalence of onchocerciasis was calculated using a random-effects model at 95% confidence interval (CI). Heterogeneity between the studies was assessed using Cochrane's Q test, P-value, I2 and visual inspection of the funnel plot. Heterogeneity was declared if P-value <0.1. The level of statistical significance for all tests was set at P-value < 0.05. Quality of the studies was checked by Newcastle-Ottawa Scale adapted for cross-sectional study. To minimize the risk of bias, publication bias was checked by drawing a funnel plots and by computing Begg and Mazumdar rank correlation and Egger's regression asymmetry test. Significant publication bias was considered if P-value < 0.05 in those tests. Presence of outlier study results was checked by leave-one-out analysis.

## Results

### Characteristics of the studies

A total of 112 studies were identified from PubMed, Google Scholar, Scopus, ScienceDirect, MEDLINE databases, and manual search. Forty-one studies were screened and recorded after duplication were removed. Finally, 21 studies were eligible after full text assessment and included in the qualitative analysis (Fig 1).

A total of 14,983 participants were recruited in the eligible studies. The smallest and largest number of participants among the studies was 83 and 1567, respectively^12^,^15^. Among the studies included, 9^16^-^24^ and 7 3,25-30 were from Oromia and SNNPR, respectively, while 2 studies were retrieved from each of Amhara^11^,^15^ and Benishangul-gumuz^12^,^31^ regions. The highest prevalence of onchocerciasis (80.5%) was recorded in Teppi town, southwest Ethiopia, among indigenous people^32^. Similarly, a prevalence of 77.6% was recorded in each of the two separate studies conducted among people residing in coffee plantation farms in Teppi^27^ and Baya Farm coffee plantation workers in Teppi town^29^. On the contrary, a very low prevalence of onchocerciasis was reported among community dwellers of Assosa (1.6%)^12^, among participants in an endemic area of Ethiopia (3.6%)^33^, and in Bench Maji Zone, southwest Ethiopia (6.3%)^25^ (Table 1).

**Table 1:** Prevalence of Onchocerca volvulus in Ethiopia from the year 1973 to 2022

No	First Author	Year of Publication	Region	Skin snip detection of *O. volvulus*			References
				Total examined [N]	Pos [N]	P [95%CI] [%]	
1	Gebrezgabiher	2020	Benishangul-Gumuz	1567	25	1.6 [1.1-2.4]	12
2	Kifle	2019	SNNPR	553	35	6.3 [4.6-8.7]	25
3	Samuel	2016	Oromia	971	395	40.7 [37.6-43.8]	16
4	Thiele	2016	Ethiopia	500	18	3.6 [2.3-5.6]	33
5	Dana	2015	Oromia	440	99	22.5 [18.9-26.6]	17
6	Dori	2011	Oromia	1114	833	74.8 [72.2-77.2]	18
7	Legesse	2010	SNNPR	390	87	22.3 [18.5-26.7	26
8	Enk	2003	Amhara	83	40	48.2 [37.8-58.9	15
9	Mengistu	2002	SNNPR	308	248	80.5 [75.7-84.6]	32
10	Hailu	2002	SNNPR	1619	1256	77.6 [75.5-79.5]	27
11	Taye	2000	SNNPR	228	39	17.1 [12.8-22.5]	28
12	Adugna	1996	Benishangul-Gumuz	931	181	19.4 [17.0-22.1]	31
13	Aga	1995	Oromia	202	110	54.5 [47.6-61.2]	19
14	Jira C	1993	Oromia	493	169	34.3 [30.2-38.6]	20
15	Workneh	1993	SNNPR	196	152	77.6 [71.2-82.8]	29
16	Bulto	1990	Oromia	826	438	53.0 [49.6-56.4]	21
17	Yeneneh	1989	Oromia	471	116	24.6 [21.0-28.7]	22
18	Gudersen	1988	Oromia	477	182	38.2 [33.9-42.6]	23
19	Tatichef	1987	SNNPR	1611	498	30.9 [28.7-33.2]	30
20	Zein ZA	1986	Amhara	1370	267	19.5 [17.5-21.7]]	11
21	Eyck DRT	1973	Oromia	633	173	27.3 [24.0-30.9]	24
	**Total**			**14983**	**5361**	**35.8 [35.0-36.6]**	

*SS = Skin snip, N = number, CI = confidence interval

### Prevalence of O. Volvulus microfilaridermia

**Fig 2:**
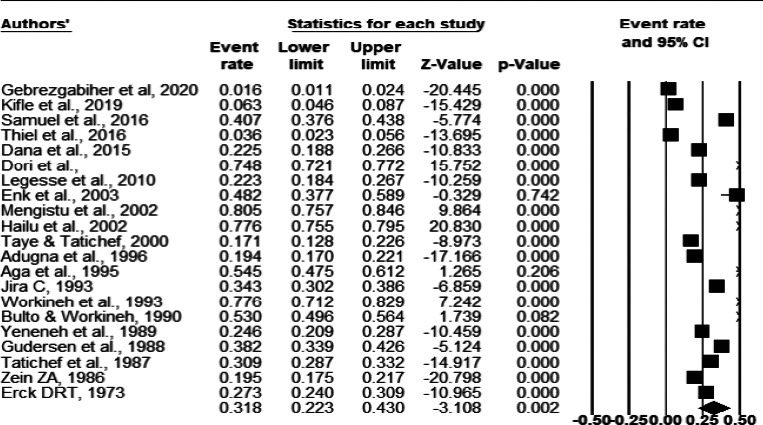
Forest plot of the prevalence of onchocerciasis in Ethiopia using random effect model

**Fig 3:**
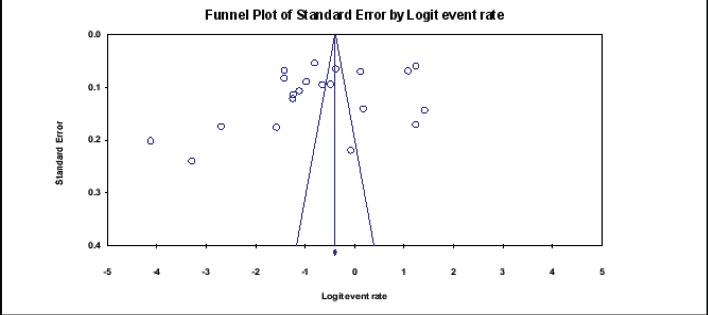
Detection of the bias of the studies was conducted using funnel plot, Begg and Mazumdar rank correlation (P-value = 0.334) and Egger's regression (P-value = 0.138)

Among 14,983 study participants, 5361 were positive for O. volvulus microfilaridermia, yielding a total prevalence of 35.8% (95%CI: 35.0-36.6). Using random effect analysis, the pooled prevalence of onchocerciasis in Ethiopia was 31.8% (95%CI: 22.3 - 43.0). The heterogeneity was high (Q = 2881.2, I2 = 99.3%, P-value < 0.001) (Fig 2). Analysis with stepwise removal of each study revealed a pooled prevalence between 29.5% and 35.5% showing no outlier results. The studies were distributed symmetrically about the combined effect size that showed the absence of publication bias (Fig 3).

### Subgroup analysis

Twenty studies have specifically reported the region where the data was collected, of which 9 (45%) and 7 (35%) were conducted in Oromia and SNNPR regions, respectively. High number of participants were also recruited in Oromia (5627, 38.9%) and SNNPR (4905, 33.9%) regions (Table 2).

**Table 2:** The prevalence of onchocercisis across regions of Ethiopia

Name of the region	Number of studies [N]	Total examined [N]	*SS* Positive [N]	Pooled prevalence (95%CI)
**Amhara**	2	1453	307	21.1 [19.1-23.3]
**Benishangul-Gumuz**	2	2498	206	8.2 [7.2-9.4]
**Oromia**	9	5627	2515	44.7 [43.4-46.0]
**SNNPR**	7	4905	2315	47.2 [45.8-48.6]
**Total**	**20**	**14483**	**5233**	**36.6 [35.9-37.4]]**

**Fig 4:**
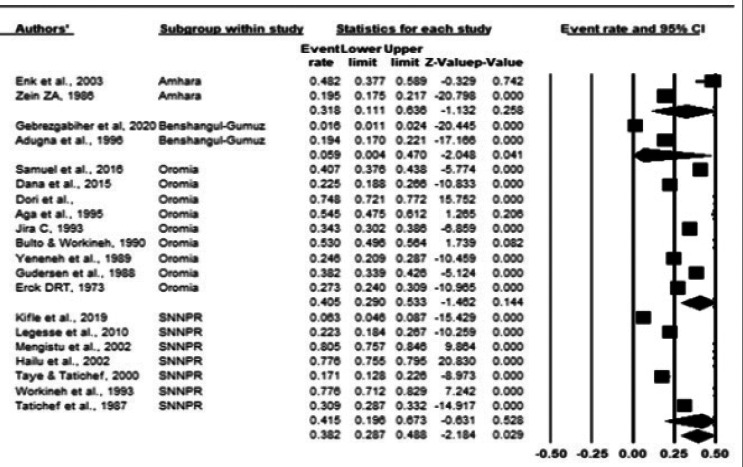
Forest plot of the prevalence of onchocerciasis across regions using random effect model

Among the regions, higher pooled prevalence of onchocerciasis was reported 41.5% (95%CI: 19.0-67.3) in SN-NPR followed by 40.5% (95%CI: 29.0-53.3) in Oromia region. Lowest pooled prevalence among regions was reported in Benishangul-Gumuz (5.9%; 95%CI: 0.4-47.0) (Fig 4).

**Fig 5:**
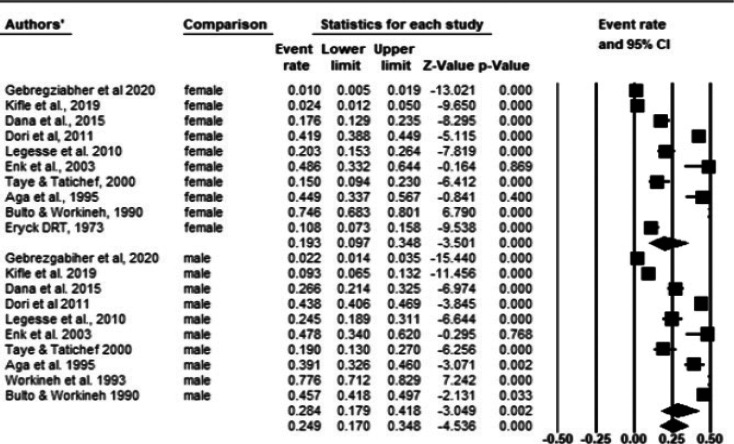
Frost plot of the prevalence of onchocerciasis across sexes using random effect model

Ten out of 21 studies have reported the number of male and female participants and their test results separately. Subgroup analysis by sex revealed that the pooled prevalence among males (28.4%; 95%CI: 17.9-41.8) and females (19.3%; 95%CI: 9.7-34.8) was significantly different (P-value < 0.001). The overall pooled prevalence of onchocerciasis across sex was 24.9% with high heterogeneity (Q-value=881.0, I2=97.8%, P-value < 0.001) (Fig 5).

**Fig 6:**
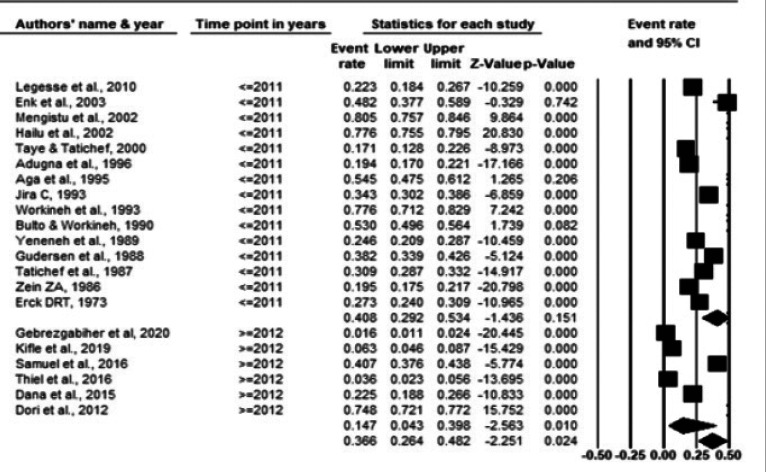
Forest plot of the prevalence of onchocerciasis before and after 2012 using random effect model

The pooled prevalence of onchocerciasis among studies conducted before and after 2012 were 40.8% and 14.7%, respectively (Fig 6). The overall pooled prevalence of onchocerciasis across publications before and after 2012 was 26.6% with high heterogeneity (Q-value=2881.2, I2=99.3%, P-value < 0.001) (Fig 6).

## Discussion

The western part of Ethiopia, where many rivers with vegetation provide a suitable habitat for the Simulium black fly vector, is known to be endemic for onchocerciasis on the basis of rapid epidemiological mapping^3^. Accordingly, 188 districts in Benishangul-Gumuz, Amhara, Oromia, SNNPR, and Gambella regions were identified as endemic for onchocerciasis. Since 2012, the country has been implementing onchocerciasis elimination strategies targeting all endemic districts. One hundred eighty-four districts were eligiblfor bi-annual treatment based on the criteria set by the Ethiopian Onchocerciasis Elimination Expert Advisory Committee in 2014 to ensure elimination by 2020^4^,^5^.

The present systematic review and meta-analysis was designed to generate comprehensive data about the national prevalence of onchocerciasis in Ethiopia. The findings provide useful epidemiological data to aid in the control of the disease. The overall pooled prevalence of O. volvulus in the present review (31.8%) was lower than findings ranging from 45.2% to 94.5% in different states of Nigeria^34^-^45^. Differences in endemicity and sex of participants might contribute to such variations. For instance, a study from Taraba State, Nigeria, recruited only males who are considered at higher risk of infection than females in many ways^36^.

On the other hand, the pooled prevalence of onchocerciasis in the present study was higher than previous findings of 5.9% from Togo^46^, 6.14% from Ghana^47^ and 2% from Mozambique^48^. Duration and level of implementation of the onchocerciasis control program have brought this difference. In Togo, endemic territories had been part of the initial onchocerciasis control program anti-vectorial intervention areas since 1976, and the vector control measures were supplemented by MDA with ivermectin since 1988^46^. In contrast to this, the national onchocerciasis control program in Ethiopia has been implemented only for about two decades period^5^. The study in Ghana was conducted in communities with a known hypoendemic distribution based on the criteria used by the Onchocerciasis Elimination Program for the Americas, where nodule and microfilarial prevalence of ≤20%, 21–59% and ≥60% are defined as hypoendemic, mesoendermic and hyperendemic, respectively^47^,^49^. Moreover, data for the study in Ghana was collected after implementation of ≥18 rounds of MDA, which contributes to lower prevalence^47^.

Region-wise analysis revealed that the pooled prevalence of onchocerciasis was highest in SNNPR (41.5%) followed by Oromia (40.5%). Majority of the studies in SNNPR were conducted at the largest coffee plantations located around Teppi. The area is known for dense vegetation accompanied by fast-flowing rivers making a conducive environment for the survival and breeding of the blackfly vector. Similarly, almost all reviewed studies from Oromia were conducted in the western part of the region where the ecology is readily suitable for the vector^16^-^30^,^32^.

There is a scarcity of available studies in the remaining endemic regions of Amhara and Benishangul-gumuz. As a result, the present review might not represent the true distribution of onchocerciasis in those regions. The pooled prevalence of the disease in Amhara region (31.8%) corresponds to mesoendemic transmission^49^. Data for both studies included in the present review were collected in northwest Ethiopia. However, more intense transmission is expected in the western parts of the region along the Benishangul-gumuz and Sudan borders^5^. Despite the endemicity of onchocerciasis in the whole region, the pooled prevalence of the disease was very low in Benishangul-Gumuz region (5.9%). The possible justifications could be: 1) only two studies were eligible and reviewed, which might not represent the regional prevalence; 2) despite people with age ≥15 are considered at risk, one of the reviewed studies in the region has included people at the age of >5 years old (194 participants out of 931 were 5-14 years-old) which could decrease the prevalence; 3) both studies in the region were conducted in Assosa, an urban setting with less vegetation compared to other districts in the region.

Simulium black flies have non-periodic feeding habits and population groups who frequently travel to or stay around the river breeding sites are at higher risk of acquiring the infection. Hence, in the present review, the pooled prevalence was higher among males than females (28.4% vs 19.3%, P-value < 0.001). This could be due to the fact that males frequently work as daily laborers in coffee plantations in Ethiopia, where the majority of infections occur. This finding was supported by studies from Mozambique^48^ and Ethiopia^50^. Contrasting results were reported in Ghana where a higher prevalence of microfilaridermia was seen in females (5.17%) relative to males (2.44%). However, the difference was not significant (P-value = 0.280)^47^.

Subgroup analysis by year of data collection resulted in a significant difference in pooled prevalence between studies conducted before (40.8%) and after (14.7%) 2012 (when the national MDA was started). This is justifiable that the national MDA with ivermectin has an impact on reducing disease transmission. However, only a few (5 out of 21) studies conducted after the start of the MDA were included in the present review, making it difficult to give a definitive conclusions.

## Conclusion

The prevalence of onchocerciasis is high in endemic areas of Ethiopia, especially in the SNNPR. Onchocerciasis is more common among males than females. The prevalence of onchocerciasis decreases after the implementation of the national MDA in Ethiopia. Therefore, the existing MDA program should be strengthened in vector control activities and creating community awareness in endemic areas.
